# Long-Term Changes in Chili Pepper Consumption and Inflammatory Biomarkers: A Longitudinal Analysis in the Moli-Sani Study

**DOI:** 10.3390/cimb48090890

**Published:** 2026-08-31

**Authors:** Claudio Tabolacci, Augusto Di Castelnuovo, Simona Costanzo, Chiara Cerletti, Amalia De Curtis, Mariarosaria Persichillo, Maria Luisa Scattoni, Mauro Biffoni, Maria Benedetta Donati, Giovanni de Gaetano, Francesco Facchiano, Licia Iacoviello, Marialaura Bonaccio

**Affiliations:** 1 Department of Oncology and Molecular Medicine, Istituto Superiore di Sanità, 00161 Rome, Italy; claudio.tabolacci@iss.it (C.T.); mauro.biffoni@iss.it (M.B.); francesco.facchiano@iss.it (F.F.); 2 Research Unit of Epidemiology and Prevention, IRCCS NEUROMED, 86077 Pozzilli, Italy; augusto.dicastelnuovo@moli-sani.org (A.D.C.); simona.costanzo@moli-sani.org (S.C.); chiara.cerletti@moli-sani.org (C.C.); amalia.decurtis@moli-sani.org (A.D.C.); mariarosaria.persichillo@moli-sani.org (M.P.); mbdonati@moli-sani.org (M.B.D.); giovanni.degaetano@moli-sani.org (G.d.G.); marialaura.bonaccio@moli-sani.org (M.B.); 3 Research Center in Epidemiology and Preventive Medicine (EPIMED), Department of Medicine and Surgery, University of Insubria, 21100 Varese, Italy; 4 National Center for Rare Diseases, Istituto Superiore di Sanità, 00161 Rome, Italy; marialuisa.scattoni@iss.it; 5 Department of Medicine and Surgery, LUM University, 70010 Casamassima, Italy

**Keywords:** chili pepper, capsaicin, cytokines, chemokines, inflammatory markers

## Abstract

Chili pepper consumption has been linked to lower cardiovascular disease (CVD) risk, although underlying mechanisms remain unclear. We investigated the association between long-term changes in chili pepper consumption and changes in circulating inflammatory markers. This longitudinal analysis included 573 adults (≥35 years) from the Italian Moli-sani Study with dietary, health, and biological data collected at baseline (2005–2010) and follow-up (2017–2020). The exposure was the 12.7-year change in chili pepper consumption frequency. Outcomes included changes in serum concentrations of C-reactive protein, eight other inflammatory factors (IL-6, IL8, IL-10, TNF-α, IFN-γ, MCP-1, IP-10, and VEGF), and the INFLA-score. Multivariable-adjusted linear regression models accounted for changes in dietary and lifestyle factors. Overall, 41.2%, 20.6%, and 38.2% of participants reduced, increased, or maintained their chili pepper consumption, respectively. A 1-unit increase in the delta of chili pepper consumption was associated with lower serum TNF-α concentrations (β = −0.85; 95% CI: −1.67, −0.03; *p* = 0.041). No associations were observed with the other inflammatory markers, although most showed downward trends. Among women, increased consumption was associated with different MCP-1 and IP-10 levels. An increase in chili pepper consumption over 12.7 years was associated with lower circulating TNF-α concentrations in this population. Although no consistent associations were observed across the inflammatory markers evaluated, these findings suggest a potential modulation of selected inflammatory pathways by sustained chili pepper consumption and warrant confirmation in larger prospective studies.

## 1. Introduction

Increasing evidence highlights the close interplay between immunity and metabolism, with nutritional status and quality of diet modulating metabolic pathways involved in immune cell function [1]. Furthermore, plant-derived bioactive compounds have been suggested to provide beneficial effects in preventing or mitigating metabolic disorders and stress-related diseases [2]. In this context, the chili pepper (*Capsicum* spp.), a plant belonging to the Solanaceae family and widely consumed worldwide as a spice and food ingredient, represents an important source of bioactive compounds. *Capsicum* fruits are a good source of flavonoids (mainly quercetin and luteolin), capsaicinoids, carotenoids, and vitamins [3]. In particular, capsaicinoids, alkaloid compounds exclusively found in the *Capsicum* genus, are responsible for the pungency of the spice and include capsaicin (CAP), dihydrocapsaicin, nordihydrocapsaicin, and homocapsaicin [4]. CAP has traditionally been used as an analgesic agent [5], but several studies showed its beneficial effects related to insulin sensitivity, glycaemia regulation, and antimicrobial activities [3,6,7]. This alkaloid possesses well-known anti-inflammatory properties [6,7], inhibiting the secretion of pro-inflammatory cytokines such as interleukin-6 (IL-6) and tumor necrosis factor α (TNF-α) in a wide range of cellular models [8,9].

CAP exerts its biological effects primarily through activation of the transient receptor potential vanilloid type 1 (TRPV1), a non-selective cation channel that induces depolarization in several cell types and increases thermogenesis, enhances fat oxidation, improves glucose metabolism, and promotes energy expenditure [6,7,10]. Although observational studies suggest a significant association between higher chili intake and a lower risk of obesity [11,12], other studies have reported that spicy food intake may promote lipid accumulation and gut microbiota dysbiosis, suggesting that its metabolic effects may vary across different geographical environments [13].

Prior longitudinal analyses in the large Moli-sani Study cohort showed that regular consumption of chili pepper was associated with a lower risk of total and cardiovascular disease (CVD) death [14]. These results were also confirmed in later epidemiological studies [15,16,17]. However, the biological mechanisms underlying these associations have not been fully elucidated, and the observational nature of many studies limits causal interpretation [18]. Furthermore, the potential effects of chili pepper consumption remain debated, considering the reported inverse associations with gastrointestinal cancers [18].

Chronic low-grade inflammation plays a key role in CVD development and progression [19,20]. However, despite the well-established anti-inflammatory properties of CAP demonstrated in experimental models [6,7], evidence from human studies investigating how chili pepper consumption influences circulating inflammatory biomarkers remains limited. Therefore, this study examined the association between long-term changes in chili pepper consumption and changes in circulating inflammatory markers in a subset of 573 participants from the Moli-sani Study, aiming to provide insight into potential biological pathways linking chili pepper intake to human health.

## 2. Materials and Methods

### 2.1. Study Population

The Moli-sani Study is a prospective cohort study established between 2005 and 2010, enrolling 24,325 men and women (aged ≥35 years) randomly selected from the general population of Molise, a region in Southern Italy, to examine risk factors for cancer, cardiovascular, and cerebrovascular diseases. Exclusion criteria included pregnancy at the time of recruitment, mental impairments, current poly-traumas or coma, and refusal to provide informed consent. Further details of the study are available elsewhere [21].

Between 2017 and 2020, a total of 5438 participants, originally recruited from the 19,211 residents of Campobasso (the capital of the Molise region) and nearby villages, were invited for a comprehensive re-examination. This included assessments of lifestyle, diet, psychological factors, medical evaluations, and blood sample collection. Of those invited, 3896 participants agreed to take part, resulting in an acceptance rate of 71.6%. Re-examinations were completed for 2581 participants between May 2017 and early March 2020. The remaining 1315 who had consented were not re-examined due to COVID-19 restrictions implemented from March 2020 onward [22]. Among those re-examined, 599 had available serum chemokine and cytokine data, and 573 were ultimately included in the final analysis after exclusions (Supplementary Figure S1).

The Moli-sani Study adheres to the Declaration of Helsinki and was approved by the Ethics Committee of the Catholic University Medical School in Rome, Italy. The re-examination was authorized by the Ethics Committee of the IRCCS Neuromed in Pozzilli (IS), Italy. All participants provided written informed consent at both baseline and re-examination.

### 2.2. Dietary Assessment

Participants’ dietary intake during the preceding 12 months was ascertained at baseline and at re-examination using an interviewer-administered semi-quantitative EPIC (European Prospective Investigation into Cancer and Nutrition) food frequency questionnaire (FFQ) that was validated for assessing energy, nutrient intake, and food group consumption in the Italian population [23]. The FFQ contains 14 sections (i.e., pasta/rice, soup, meat [excluding salami and other cured meats], fish, raw vegetables, cooked vegetables, eggs, sandwiches, salami and other cured meats, cheese, fruit, bread/wine, milk/coffee/cakes, and herbs/spices) with 248 questions concerning 188 different food items [23]. Using a specifically designed software, frequencies and quantities of each food were linked to Italian Food Tables [24] to obtain estimates of daily intake of macro- and micro-nutrients plus energy. At both baseline and re-examination, the frequency of chili pepper intake was estimated through the question: ‘How often do you consume foods containing chili pepper?’, with possible answers being ‘never or almost never’, ‘occasionally’, ‘often’, or ‘very often’. Based on the reported frequency, chili pepper consumption was expressed as the number of consumption occasions per week. For the analyses, chili pepper was categorized as never/rare intake (0 times/week), >0 to ≤2 times/week, >2 to ≤4 times/week, and >4 times/week, and the four categories were assigned scores of 0, 1, 2, and 3, respectively. Adherence to the traditional Mediterranean Diet (MD) was assessed using the MD score (MDS) developed by Trichopoulou and colleagues [25].

### 2.3. Blood Collection and Biomarker Measurements

Venous blood samples were collected from participants who had fasted overnight and abstained from smoking for at least 6 h. Serum chemokines/cytokines and growth factor levels were measured on both baseline and re-examination biological samples, all stored in vapor liquid nitrogen at the Neuromed Biobanking Centre (Pozzilli, Italy). In detail, IL-6, IL-8 (CXCL8), IL-10, TNF-α, vascular endothelial growth factor (VEGF), monocyte chemoattractant protein-1 (MCP-1/CCL2), interferon gamma-induced protein 10 (IP-10/CXCL10), and interferon-γ (IFN-γ) were measured using xMAP technology and a customized assay (Human Luminex^®^ Discovery Assay, R&D Systems, Minneapolis, MN, USA) according to the manufacturer’s instructions. The quantification was carried out with Bio-Plex 200 System reader (BioRad, Hercules, CA, USA) equipped with a magnetic workstation and Bio-Plex ManagerTM software (Version 6.1). Results are expressed as pg/mL. Missing values (<5% of observations for all markers), were imputed through the k-nearest neighbor algorithm (kNN() function, k = 20), of the VIM package [26] in R version 4.0.2 (accessed on 11 October 2020) [27].

Serum high sensitivity C-reactive protein (CRP) was measured by a particle-enhanced immunoturbidimetric assay (ILab 350, Instrumentation Laboratory, Milan, Italy at baseline, and ILab Aries at re-examination). Quality control was assured using commercial (high and low) laboratory standards. Hemochromocytometric analysis was performed by cell count (Coulter HMX, Beckman Coulter, IL Milan, Italy at baseline, and Siemens ADVIA 120 at re-examination) within 3 h from blood collection. Since the baseline measurements of neutrophil percentage were largely missing, granulocyte percent was considered as an accurate predictor [28]. The granulocyte to lymphocyte ratio (GLR) was therefore calculated. The same procedures were used for collecting data during the re-examination of the cohort.

A low-grade inflammation score (INFLA-score) was computed by summarizing the synergistic effects of 4 inflammatory biomarkers, as previously described in the Moli-sani Study cohort [28,29,30]. The INFLA-score was calculated for both baseline and re-examination samples. Briefly, deciles of each biomarker level (CRP, WBC, platelets, granulocyte % to lymphocyte % ratio (GLR)) were generated. For all four components, being in the highest deciles (7 to 10) gave a score which increased from 1 to 4, while being in the lowest deciles (1 to 4) was negatively scored from −4 to −1. Being in the deciles 5 or 6 resulted in zero points. Therefore, reflecting the sum of the four biomarkers, the INFLA-score ranged from −16 to 16. An increase in the score represented an increase in low-grade inflammation intensity.

### 2.4. Assessment of Covariates

Data on socio-demographic, lifestyle, and clinical variables were self-reported and collected through interviewer-administered questionnaires, using the same standardized procedures at both baseline and re-examination visits.

Education level was categorized according to the highest qualification attained: up to lower secondary (≤8 years of schooling), upper secondary school (>8 ≤13 years), and postsecondary (>13 years). Occupational class was classified as professional/managerial, skilled non-manual, skilled manual, semi-skilled/unskilled, and unemployed/unclassified, based on the Registrar General’s occupational classification system. Housing tenure was categorized as rented, single dwelling ownership, and multiple dwelling ownership. Marital status was coded as married/cohabiting, divorced/separated, single/unmarried, or widowed.

Leisure-time physical activity was assessed using a structured questionnaire administered by the interviewer [31] and expressed as daily energy expenditure in metabolic equivalent task-hours (MET-h/d) for sports, walking, and gardening. Changes in leisure-time physical activity levels were determined by subtracting baseline values from those at the 12.7-year re-examination. Participants were classified as never, current, or former smokers, depending on whether they had smoked in the past 12 months. Height and weight were measured, and body mass index (BMI) was calculated as kg/m^2^.

History of cardiovascular disease (including angina, myocardial infarction, revascularization procedures, peripheral artery disease, and cerebrovascular events) was self-reported and confirmed through medical records and therapy documentation. A history of cancer was also self-reported and confirmed by medical records. Participants were considered to have diabetes, hypertension, or hyperlipidaemia if they were receiving medications specific to those conditions.

### 2.5. Statistical Analysis

In this analysis, the exposure variable was the 12.7-year change in chili pepper consumption, while the outcomes were the corresponding changes in serum concentrations of chemokines/cytokines over the same 12.7-year period. Change in chili pepper consumption was calculated as the difference between the chili pepper consumption score at re-examination and the score at baseline (follow-up minus baseline). Since the score ranged from 0 to 3, the resulting delta score theoretically ranged from −3 to +3.

Thus, a positive delta value indicated an increase in chili pepper consumption over time, a negative value indicated a decrease, and a value of zero indicated no change in the consumption category.

The delta score was analyzed as a continuous variable. Although chili pepper consumption was assessed on an ordinal scale, adjacent categories were assumed to represent approximately equal increments in consumption, allowing the delta score to be modeled continuously to evaluate dose–response associations. Effect estimates were expressed per 1-point increase in the delta score, corresponding to a one-category increase in chili pepper consumption over follow-up.

For additional analyses, the delta score was categorized into three groups: decreased consumption (delta < 0), stable consumption (delta = 0; reference category), and increased consumption (delta > 0).

Multivariable linear regression analysis (PROC REG in SAS) was used to estimate the relationship between changes in chili pepper consumption (modeled both as categorical and continuous variable) and changes in inflammatory markers. Results were expressed as regression coefficients (β) with 95% confidence intervals (95% CI). Potential confounders were defined based on existing literature rather than statistical criteria and were identified due to their documented associations with both diet and inflammatory markers.

Adjustments were made for baseline age, sex, duration of re-examination, educational level, baseline chili consumption, baseline MDS and changes in MDS, baseline energy intake and changes in energy intake, history of CVD (at baseline and re-examination), history of cancer (at baseline and re-examination), use of medication for diabetes (at baseline and re-examination), use of medication for high blood pressure (at baseline and re-examination), use of lipid-lowering drugs (at baseline and re-examination), baseline levels of leisure-time physical activity and changes in leisure-time physical activity, baseline BMI and changes in BMI, smoking status (at baseline and re-examination), menopausal status (at baseline and re-examination), and baseline levels of each inflammatory marker.

Given the exploratory nature of the analysis, which aimed to investigate whether long-term changes in chili pepper consumption were associated with alterations in a panel of circulating inflammatory biomarkers, no adjustment for multiple comparisons was applied. The evaluated biomarkers were considered as interrelated components of the same inflammatory pathway, and results were interpreted cautiously as hypothesis-generating. Multiple imputation was performed under a missing-at-random assumption to reduce the loss of information due to missing covariate data. Analyses were conducted for the entire sample, as well as separately for men and women. Data analysis was performed using SAS/STAT software, version 9.4 (SAS Institute Inc., Cary, NC, USA).

## 3. Results

The study population of 573 participants was followed up for a median of 12.7 y (IQR= 12.5 − 12.9 y), and the mean age at baseline was 52.3 ± 9.3 y (min-max 35.6–75.7 y). At the 12.7-year re-examination, the proportion of participants with reduced, stable, and increased chili pepper consumption was 41.2%, 38.2%, and 20.6%, respectively (Table 1). The mean change (delta) in chili pepper consumption was −0.36 (SD = 1.26; times/week), with values ranging from −3 to +3.

Participants who increased their chili pepper consumption over time were, on average, older at re-examination and reported greater increases in physical activity levels compared with those who reduced their chili pepper intake (Table 1). Other baseline characteristics were not associated with changes in chili pepper consumption over time. Mean serum concentrations of analytes at baseline and re-examination are shown in Supplementary Table S1.

In multivariable-adjusted analyses, a 1-unit increase in the delta of chili pepper consumption (reflecting an increase in consumption over time) was associated with lower serum TNF-α concentrations (β = −0.85; 95% CI: −1.67, −0.03; *p* = 0.041; Table 2). A similar inverse association was observed for IL-10 concentrations (β = −7.95; 95% CI: −16.36, 0.46; *p* = 0.064; Table 2). No associations were observed between changes in chili pepper consumption and the remaining inflammatory markers or the INFLA-score.

Among women, a 1-unit increase in the delta of chili pepper consumption was associated with higher serum MCP-1/CCL2 concentrations (β = 14.14; 95% CI: 1.53, 26.75; *p* = 0.028) and lower IP-10/CXCL10 levels (β = −14.00; 95% CI: −24.57, −3.42; *p* = 0.010) (Table 3).

These associations were not observed among men (Table 4). No associations were found between changes in chili pepper consumption and the other inflammatory markers evaluated in either sex.

## 4. Discussion

Metabolic syndrome is a cluster of conditions and risk factors, including obesity, insulin resistance, dyslipidaemia, and hypertension, that increase the risk of diabetes, CVD, cancers, and other human diseases related to lifestyle [32]. In particular, CVDs remain the leading cause of death worldwide, especially in people with metabolic disorders [33]. The obesity-induced inflammation is driven by the secretion of pro-inflammatory cytokines/chemokines like TNF-α, MCP-1/CCL2, and IL-6, among others by adipocytes [34]. The consumption of large quantities of fruits and vegetables is important for the health effects due to their antioxidant and anti-inflammatory capabilities, and this is crucial for reducing the incidence of metabolic syndrome [35].

Among plant-derived foods, chili pepper has attracted considerable interest because of its widespread consumption and its content of bioactive compounds, including capsaicinoids, flavonoids, carotenoids, and vitamins [7]. A substantial body of evidence has supported the potential benefits of chili pepper in protecting against various metabolic complications and CVD [10,14]. To the best of our knowledge, studies specifically investigating the association between chili pepper intake and circulating inflammatory mediators are limited. However, our results indicated that an increase in chili pepper consumption over time was associated with lower circulating TNF-α levels, whereas no associations were observed for the other inflammatory markers evaluated. As an inflammatory cytokine with pleiotropic effects, TNF-α represents a major regulator of the inflammatory immune response, involved in numerous autoimmune diseases or chronic inflammatory pathologies [36,37,38]. Our findings are in line with previous experimental studies suggesting that *Capsicum* extracts may reduce TNF-α production in both in vitro and in vivo models [39,40]. Interestingly, the effects induced by chili pepper are comparable to those obtained from the use of CAP alone [8,9]. The reported anti-inflammatory properties of CAP have been related to its ability to inhibit the NF-κB and MAPK/ERK signaling pathway, resulting in reduced expression and secretion of pro-inflammatory cytokines such as TNF-α and IL-6 [41]. Moreover, in vitro evidence suggests that an inflammatory response may be modulated through activation of TRPV1, whose activation appears to regulate the β-defensin and NF-κB signaling pathways [42]. The immunomodulatory role of CAP has been highlighted by several in vivo experimental studies, supporting the biological plausibility of our observations, although the protocols of CAP administration used in animal models may not directly correspond to habitual dietary chili pepper consumption in humans. For example, in mouse models of lipopolysaccharide (LPS)-induced inflammation, CAP has been shown to modulate systemic inflammatory responses by reducing the levels of IL-6 and MCP-1/CCL2 while increasing the production of IL-10 through activation of the TRPV1 pathway [43]. Consistent with these findings, CAP administration in LPS-treated mice reduced both TNF-α mRNA expression and circulating TNF-α levels, suggesting that CAP may attenuate TNF-α production at both the transcriptional and post-transcriptional levels [44]. Interestingly, CAP supplementation in apolipoprotein E (ApoE)-deficient mice reduced systemic inflammation and atherosclerotic lesions with lipid-lowering effects, mainly through the activation of TRPV1 and PPARγ signaling [45]. Although a trend toward lower circulating IL-10 levels, an immunomodulatory cytokine, was observed with increasing chili pepper intake, the biological relevance of this finding remains unclear. This finding suggests that further studies are needed to clarify whether chili pepper intake influences the circulating levels of other cytokines.

Sex-stratified analyses suggested potential differences in the association between changes in chili pepper consumption and serum cytokine/chemokine levels. Specifically, in women, but not in men, an increase in chili pepper consumption was associated with higher serum MCP-1/CCL2 levels and lower IP-10/CXCL10 levels, two chemokines strongly involved in inflammatory processes [46]. These findings may suggest a potential modulation of selected immune signaling pathways, involving chemokines related to monocyte/macrophage recruitment and IFN-γ-mediated T-cell responses, although the underlying mechanisms remain unclear. Although data regarding the effects of chili pepper intake and MCP-1 and IP-10 levels in human samples are lacking, our findings appear to be in contrast with the observation that in obese mice, CAP inhibited the expressions and release of IL-6 and MCP-1 [34]. On the contrary, it has been demonstrated that CAP positively affects skin wound healing through the reduction of IP-10 levels, among others [47]. However, gender differences and the effects of chili pepper consumption warrant further investigation, particularly given that the perception and preference of spicy foods differ between men and women, with women tending to prefer less spicy foods [48]. Moreover, accumulating experimental evidence indicates that estrogen upregulates TRPV1, highlighting a potential neuronal mechanism underlying sex differences in pain perception [49].

Short-term pepper consumption has been shown to affect gut microbiome composition [50]. Similarly, CAP-induced modifications of the gut microbiota have been proposed as another possible mechanism underlying its anti-obesity effects [51]. Consistently, CAP has been shown to attenuate LPS-induced hepatic and intestinal inflammation in mice by reducing several inflammatory markers, including TNF-α, while also modulating the composition of the colonic microbiota [52]. Therefore, future studies should consider the role of gut microbiota in interpreting the effects of spicy food consumption in human health, since microbiome-mediated pathways could represent an important component of its metabolic and immunomodulatory actions. Although TNF-α was the only biomarker showing an association with changes in chili pepper consumption in the overall population, these findings provide a biologically plausible signal that deserves further investigation. In fact, it has been demonstrated that a one-unit increase in serum TNF-α may intensify the inflammatory activity in early rheumatoid arthritis [53]. Therefore, considering the role of TNF-α as a key mediator of low-grade systemic inflammation and metabolic dysfunction [54], the observed inverse association with changes in chili pepper consumption may represent a potential pathway linking chili pepper intake with cardiometabolic health.

## 5. Strengths and Limitations

Findings from this study derive from a subset of participants in a well-established population-based cohort. A key strength is the repeated assessment of dietary and lifestyle factors over time. Unlike many epidemiological investigations that rely solely on baseline data, this approach allows for capturing individual-level changes, which are often overlooked in studies that focus on cross-sectional or group-level comparisons. Additionally, the study benefits from its population-based, long-term prospective design and the inclusion of a wide range of covariates to help control for potential confounding.

Nevertheless, several limitations should be acknowledged. As an observational study, causal relationships cannot be definitively established. Dietary intake was self-reported, which introduces the possibility of recall bias, inaccuracies in estimating portion sizes, and limitations inherent in food composition databases. These concerns were partially addressed by excluding individuals with implausible energy intake and applying energy adjustment methods [55]. The FFQ used has demonstrated predictive validity for various health outcomes in previous research [56]. Despite thorough adjustment, residual confounding due to unmeasured variables or measurement errors in covariates cannot be ruled out.

Moreover, this analysis was limited to a subset of participants who underwent follow-up after 12.7 years, and these individuals may differ systematically from the broader eligible cohort, potentially introducing selection bias and limiting the generalizability of the findings. The inflammatory markers assessed in this study were limited and did not capture the full range of molecules involved in systemic inflammation. Important mediators beyond the selected cytokines and chemokines might have been missed, potentially overlooking additional effects of chili pepper consumption. As this study was designed as an exploratory investigation of potential inflammatory pathways, results should be interpreted cautiously, considering the number of biomarkers evaluated.

Lastly, observed dietary changes may partly reflect regression to the mean or shifts related to health status over time, which should be considered when interpreting the results.

## 6. Conclusions

In this prospective analysis of an Italian adult population, an increase in chili pepper consumption over 12.7 years was associated with reduced serum levels of TNF-α, a key pro-inflammatory cytokine involved in cardiometabolic diseases. However, the observation of associations with only TNF-α in the overall population and MCP-1/CCL2 and IP-10/CXCL10 among women, together with the absence of changes in the other inflammatory markers or the INFLA-score, suggests that chili pepper consumption may influence selected immune signaling pathways rather than induce a generalized reduction of systemic inflammation. Given the observational nature of the study, the modest sample size, and the exploratory assessment of multiple inflammatory biomarkers, these findings should be considered hypothesis-generating. Further research in larger cohorts is needed to replicate these associations and clarify the underlying biological mechanisms.

## Figures and Tables

**Table 1:** Characteristics of the participants and changes over time, according to changes in chili pepper consumption after a median 12.7 y re-examination, the Moli-sani Study.

		Changes in Chili Pepper Consumption				
		All	Reduced	Relatively Stable	Increased	*p*-Value ^b^
N of participants (%)		573	236 (41.2)	219 (38.2)	118 (20.6)	–
Chili pepper consumption at baseline (times/week; mean, SD)		2.9 (3.7)	4.8 (4.2)	2.1 (2.9)	0.6 (0.9)	<0.0001
Chili pepper consumption at re-examination (times/week; mean, SD)		2.2 (4.2)	0.9 (1.0)	2.7 (5.9)	4.0 (3.6)	<0.0001
Change in chili pepper consumption (delta; times/week; mean, SD)		−0.36 (1.26)	−1.57 (0.73)	0.00 (0.00)	1.42 (0.59)	<0.0001
MD score at baseline (mean, SD) ^a^		4.86 (1.65)	5.04 (1.62)	4.79 (1.59)	4.61 (1.79)	0.25
MD score at re-examination (mean, SD) ^a^		4.34 (1.65)	4.36 (1.64)	4.36 (1.68)	4.31 (1.61)	0.86
MD score change (mean, SD) ^a^		−0.51 (2.02)	−0.68 (2.09)	−0.44 (1.98)	−0.30 (1.91)	0.68
Energy intake (kcal/d; mean, SD)		2208 (588)	2219 (594)	2223 (597)	2262 (560)	0.34
Change in energy intake (mean, SD)		−403 (597)	−438 (590)	−409 (613)	−320 (579)	0.46
Women (%)		53.6	52.5	56.6	50.0	0.47
Age at baseline (y; mean, SD) ^a^		52.3 (9.3)	53.8 (9.0)	51.0 (9.4)	51.6 (9.1)	0.0028
Age at re-examination (y; mean, SD) ^a^		65.0 (9.3)	66.6 (9.0)	63.7 (9.5)	64.3 (9.1)	0.0023
Educational level (%)						0.24
Up to lower secondary		30.4	34.7	26.9	28.0	
Upper secondary		47.8	45.3	50.7	47.5	
Postsecondary		21.8	20.0	22.4	24.5	
Housing (%)						0.31
Rent		9.4	11.9	9.6	4.2	
1 dwelling ownership		68.1	66.1	66.2	75.4	
>1 dwelling ownership		22.5	22.0	24.2	20.3	
Smoking status at baseline (%)						0.78
Non-smoker		46.4	45.8	46.6	47.5	
Smoker		25.7	25.4	25.6	26.3	
Former		27.9	28.8	27.8	26.2	
Smoking status at re-examination (%)						0.76
Non-smoker		49.7	48.3	52.0	48.3	
Smoker		15.5	14.8	16.0	16.1	
Former		34.8	36.9	32.0	35.6	
Body mass index at baseline (kg/m^2^; mean, SD)		27.0 (4.1)	27.3 (4.0)	26.6 (4.1)	27.2 (4.4)	0.40
Body mass index change (mean, SD)		0.92 (2.66)	0.89 (2.92)	1.16 (2.50)	0.55 (2.33)	0.13
Leisure-time physical activity at baseline (MET-h/d; mean, SD) ^a^		3.90 (4.28)	4.24 (4.54)	3.69(4.16)	3.60 (3.93)	0.40
Leisure-time physical activity change (MET-h/d; mean, SD) ^a^		0.33 (4.88)	−0.13 (4.93)	0.14 (4.60)	1.61 (5.09)	0.013
Cardiovascular disease at baseline (%)		3.8	5.1	3.2	2.5	0.64
Cardiovascular disease at re-examination (%)		10.1	11.9	9.1	8.5	0.83
Cancer at baseline (%)		1.8	1.7	1.8	1.7	0.96
Cancer at re-examination (%)		10.1	11.9	11.0	5.1	0.13
Medication for diabetes at baseline (%)		2.4	3.4	2.3	0.9	0.40
Medication for diabetes at re-examination (%)		10.8	11.4	7.8	15.2	0.10
Medication for high blood pressure at baseline (%)		20.9	24.2	19.6	17.0	0.53
Medication for high blood pressure at re-examination (%)		48.0	50.0	48.0	44.1	0.44
Lipid-lowering drugs at baseline (%)		6.3	6.8	5.9	5.9	0.99
Lipid-lowering drugs at re-examination (%)		20.2	24.6	16.0	19.5	0.29

SD, standard deviation; MD, Mediterranean Diet Score; MET-h, metabolic equivalents hours per day. ^a^ Means are unadjusted. ^b^ Except for age and sex, *p*-values are for the comparison across reduced/relatively stable/increased chili pepper consumption groups and were obtained from multivariable-adjusted generalized linear models including age at baseline, sex, baseline energy intake and changes in energy intake, and duration of re-examination.

**Table 2:** Changes in chili pepper consumption and changes in markers of inflammation at the end of a median 12.7 y re-examination in the Moli-sani Study cohort (*n* = 573), using data obtained by multiple imputation.

	Trajectories of Chili Pepper Consumption				
	Reduced	Stable	Increased	1-Unit Increase	*p* Value
N of subjects (%)	236 (41.2)	219 (38.2)	118 (20.6)	–	–
Δ TNF-α	0.53 (−1.61, 2.67)	Ref.	−1.24 (−3.53, 1.05)	−0.85 (−1.67, −0.03)	0.041
Δ VEGF	−9.85 (−35.69, 16.00)	Ref.	19.51 (−8.19, 47.21)	7.02 (−2.91, 16.96)	0.17
Δ MCP-1	15.07 (−16.44, 46.58)	Ref.	30.19 (−3.57, 63.96)	4.00 (−8.13, 16.13)	0.52
Δ IP-10	24.48 (−0.11, 49.07)	Ref.	−0.016 (−26.33, 26.30)	−8.18 (−17.67, 1.31)	0.091
Δ IL-6	−0.54 (−3.79, 2.70)	Ref.	1.16 (−2.31, 4.64)	0.25 (−1.00, 1.49)	0.70
Δ IL-8	0.26 (−1.50, 2.02)	Ref.	−0.04 (−1.92, 1.84)	0.016 (−0.66, 0.69)	0.96
Δ IL-10	−2.71 (−24.60, 19.17)	Ref.	−13.73 (−37.15, 9.69)	−7.95 (−16.36, 0.46)	0.064
Δ IFN-γ	−0.39 (−4.52, 3.74)	Ref.	−1.40 (−5.83, 3.02)	−0.53 (−2.11, 1.06)	0.51
Δ CRP	−0.34 (−1.42, 0.74)	Ref.	−0.68 (−1.85, 0.48)	0.07 (−0.35, 0.49)	0.74
Δ INFLA-Score	−0.39 (−1.44, 0.66)	Ref.	−0.38 (−1.50, 0.75)	−0.06 (−0.47, 0.34)	0.76

TNF-α, tumor necrosis factor α; VEGF, vascular endothelial growth factor; MCP-1, monocyte chemoattractant protein-1; IP-10, interferon gamma-induced protein 10; IL-6, interleukin-6; IL-8, interleukin-8; IL-10, interleukin-10; IFN-γ, interferon-γ; CRP, C-reactive protein. Regression coefficients (β) with 95% confidence intervals (95% CI) were obtained from multivariable linear regression models adjusted for age, sex, duration of follow-up, educational level, baseline chili pepper consumption, baseline Mediterranean diet score (MDS) and changes in MDS, baseline energy intake and changes in energy intake, history of CVD (at baseline and re-examination), history of cancer (at baseline and re-examination), use of medication for diabetes (at baseline and re-examination), use of antihypertensive medication (at baseline and re-examination), use of lipid-lowering drugs (at baseline and re-examination), baseline leisure-time physical activity and changes in leisure-time physical activity, baseline BMI and changes in BMI, smoking status (at baseline and re-examination), menopausal status (at baseline and re-examination), and baseline concentrations of the corresponding biomarker. The β coefficient reported in the last column represents the estimated change in each dependent variable per 1-unit increase in the delta of chili pepper consumption obtained from multivariable linear regression models, adjusted as described above. The independent variable was calculated as the difference between chili pepper consumption frequency at re-examination and baseline (follow-up minus baseline), with both measures categorized into four levels (0 = no intake, 1, 2, and 3 = increasing frequency categories). The delta score ranged from −3 to +3, where positive values indicated increased chili pepper consumption, negative values indicated decreased consumption, and zero indicated stable consumption.

**Table 3:** Changes in chili pepper consumption and changes in markers of inflammation at the end of a median 12.7 y re-examination amongst women from the Moli-sani Study cohort (*n* = 307), using data obtained by multiple imputation.

	Trajectories of Chili Pepper Consumption				
	Reduced	Stable	Increased	1-Unit Increase	*p* Value
N of subjects (%)	124 (40.4)	124 (40.4)	59 (19.2)	–	–
Δ TNF-α	1.21 (−3.05, 5.46)	Ref.	−1.61 (−5.69, 2.47)	−1.26 (−2.75, 0.24)	0.10
Δ VEGF	−4.02 (−40.35, 32.31)	Ref.	25.37 (−9.46, 60.21)	7.23 (−5.64, 20.10)	0.27
Δ MCP-1	11.82 (−23.10, 46.73)	Ref.	71.61 (38.19, 105.03)	14.14 (1.53, 26.75)	0.028
Δ IP-10	25.20 (−4.74, 55.113)	Ref.	−4.74 (−33.39, 23.91)	−14.00 (−24.57, −3.42)	0.010
Δ IL-6	−1.46 (−8.19, 5.27)	Ref.	2.26 (−4.20, 8.71)	0.29 (−2.08, 2.67)	0.81
Δ IL-8	0.058 (−2.22,2.34)	Ref.	0.15 (−2.02, 2.33)	−0.012 (−0.81, 0.79)	0.98
Δ IL-10	−17.92 (−54.83, 19.00)	Ref.	−31.41 (−66.80, 3.98)	−10.13 (−23.21, 2.96)	0.13
Δ IFN-γ	0.39 (−7.90, 8.68)	Ref.	−3.71 (−11.67, 4.25)	−2.00 (−4.93, 0.93)	0.18
Δ CRP	0.60 (−1.36, 2.56)	Ref.	−1.33 (−3.21, 0.55)	−0.40 (−1.10, 0.30)	0.26
Δ INFLA-Score	−0.50 (−2.11, 1.11)	Ref.	−0.93 (−2.47, 0.61)	−0.13 (−0.70, 0.44)	0.65

TNF-α, tumor necrosis factor α; VEGF, vascular endothelial growth factor; MCP-1, monocyte chemoattractant protein-1; IP-10, interferon gamma-induced protein 10; IL-6, interleukin-6; IL-8, interleukin-8; IL-10, interleukin-10; IFN-γ, interferon-γ; CRP, C-reactive protein. Regression coefficients (β) with 95% confidence intervals (95% CI) were obtained from multivariable linear regression models adjusted for age, duration of follow-up, educational level, baseline chili pepper consumption, baseline Mediterranean diet score (MDS) and changes in MDS, baseline energy intake and changes in energy intake, history of CVD (at baseline and re-examination), history of cancer (at baseline and re-examination), use of medication for diabetes (at baseline and re-examination), use of antihypertensive medication (at baseline and re-examination), use of lipid-lowering drugs (at baseline and re-examination), baseline leisure-time physical activity and changes in leisure-time physical activity, baseline BMI and changes in BMI, smoking status (at baseline and re-examination), menopausal status (at baseline and re-examination), and baseline concentrations of the corresponding biomarker. The β coefficient reported in the last column represents the estimated change in each dependent variable per 1-unit increase in the delta of chili pepper consumption obtained from multivariable linear regression models, adjusted as described above. The independent variable was calculated as the difference between chili pepper consumption frequency at re-examination and baseline (follow-up minus baseline), with both measures categorized into four levels (0 = no intake, 1, 2, and 3 = increasing frequency categories). The delta score ranged from −3 to +3, where positive values indicated increased chili pepper consumption, negative values indicated decreased consumption, and zero indicated stable consumption.

**Table 4:** Changes in chili pepper consumption and changes in markers of inflammation at the end of a median 12.7 y re-examination amongst men from the Moli-sani Study cohort (*n* = 266) using data obtained by multiple imputation.

	Trajectories of Chili Pepper Consumption				
	Reduced	Stable	Increased	1-Unit Increase	*p* Value
N of subjects (%)	95 (35.7)	112 (42.1)	59 (22.2)	–	–
Δ TNF-α	−0.26 (−1.66, 1.14)	Ref.	−1.21 (−2.88, 0.46)	−0.35 (−0.92, 0.22)	0.23
Δ VEGF	−23.61 (−63.25, 16.01)	Ref.	14.75 (−32.44, 61.95)	11.50 (−4.61, 27.61)	0.16
Δ MCP-1	−3.14 (−58.05, 51.78)	Ref.	−28.97 (−94.44, 36.51)	−3.24 (−25.54, 19.06)	0.78
Δ IP-10	8.86 (−32.76, 50.48)	Ref.	−6.29 (−55.94, 43.37)	−1.34 (−18.27, 15.59)	0.88
Δ IL-6	0.46 (−0.92, 1.85)	Ref.	−0.014 (−1.69, 1.64)	0.083 (−0.48, 0.64)	0.77
Δ IL-8	0.35 (−2.47, 3.17)	Ref.	0.38 (−2.99, 3.75)	0.40 (−0.75, 1.55)	0.50
Δ IL-10	1.93 (−24.15, 28.02	Ref.	6.33 (−24.77, 37.43)	−3.09 (−13.69, 7.52)	0.57
Δ IFN-γ	−0.46 (−1.11, 0.18)	Ref.	0.067 (−0.70, 0.84)	0.14 (−0.12, 0.41)	0.28
Δ CRP	−0.73 (−1.95, 0.49)	Ref.	0.15 (−1.29, 1.60)	0.48 (−0.02, 0.97)	0.058
Δ INFLA-Score	−0.76 (−2.27, 0.76)	Ref.	0.43 (−1.35, 2.21)	0.19 (−0.43, 0.80)	0.55

TNF-α, tumor necrosis factor α; VEGF, vascular endothelial growth factor; MCP-1, monocyte chemoattractant protein-1; IP-10, interferon gamma-induced protein 10; IL-6, interleukin-6; IL-8, interleukin-8; IL-10, interleukin-10; IFN-γ, interferon-γ; CRP, C-reactive protein. Regression coefficients (β) with 95% confidence intervals (95% CI) were obtained from multivariable linear regression models adjusted for age, duration of follow-up, educational level, baseline chili pepper consumption, baseline Mediterranean diet score (MDS) and changes in MDS, baseline energy intake and changes in energy intake, history of CVD (at baseline and re-examination), history of cancer (at baseline and re-examination), use of medication for diabetes (at baseline and re-examination), use of antihypertensive medication (at baseline and re-examination), use of lipid-lowering drugs (at baseline and re-examination), baseline leisure-time physical activity and changes in leisure-time physical activity, baseline BMI and changes in BMI, smoking status (at baseline and re-examination), and baseline concentrations of the corresponding biomarker. The β coefficient reported in the last column represents the estimated change in each dependent variable per 1-unit increase in the delta of chili pepper consumption obtained from multivariable linear regression models, adjusted as described above. The independent variable was calculated as the difference between chili pepper consumption frequency at re-examination and baseline (follow-up minus baseline), with both measures categorized into four levels (0 = no intake, 1, 2, and 3 = increasing frequency categories). The delta score ranged from −3 to +3, where positive values indicated increased chili pepper consumption, negative values indicated decreased consumption, and zero indicated stable consumption.

## Data Availability

The data underlying this article will be shared on reasonable request to the corresponding author. The data are stored in an institutional repository (https://repository.neuromed.it) (accessed on 28 October 2019), and access is restricted by the ethical approvals and the legislation of the European Union.

## Supplementary Materials

The following supporting information can be downloaded at: https://www.mdpi.com/article/10.3390/cimb48090890/s1.
