# Supplementary material for: Long-Term Changes in Chili Pepper Consumption and Inflammatory Biomarkers: A Longitudinal Analysis in the Moli-Sani Study
Source: Curr Issues Mol Biol. 2026 Aug 31;48(9):890. doi: 10.3390/cimb48090890 (PMC13605114; doi:10.3390/cimb48090890)
Supplement: Supplementary file 1 [file cimb-48-00890-s001.zip › cimb-4519260-supplementary.pdf]

Article

Long-term changes in chili pepper consumption and inflammatory biomarkers: a longitudinal analysis in the Moli-sani Study

Tabolacci C. et al.

Supplementary Materials

Figure S1. Flowchart for selection of study participants.

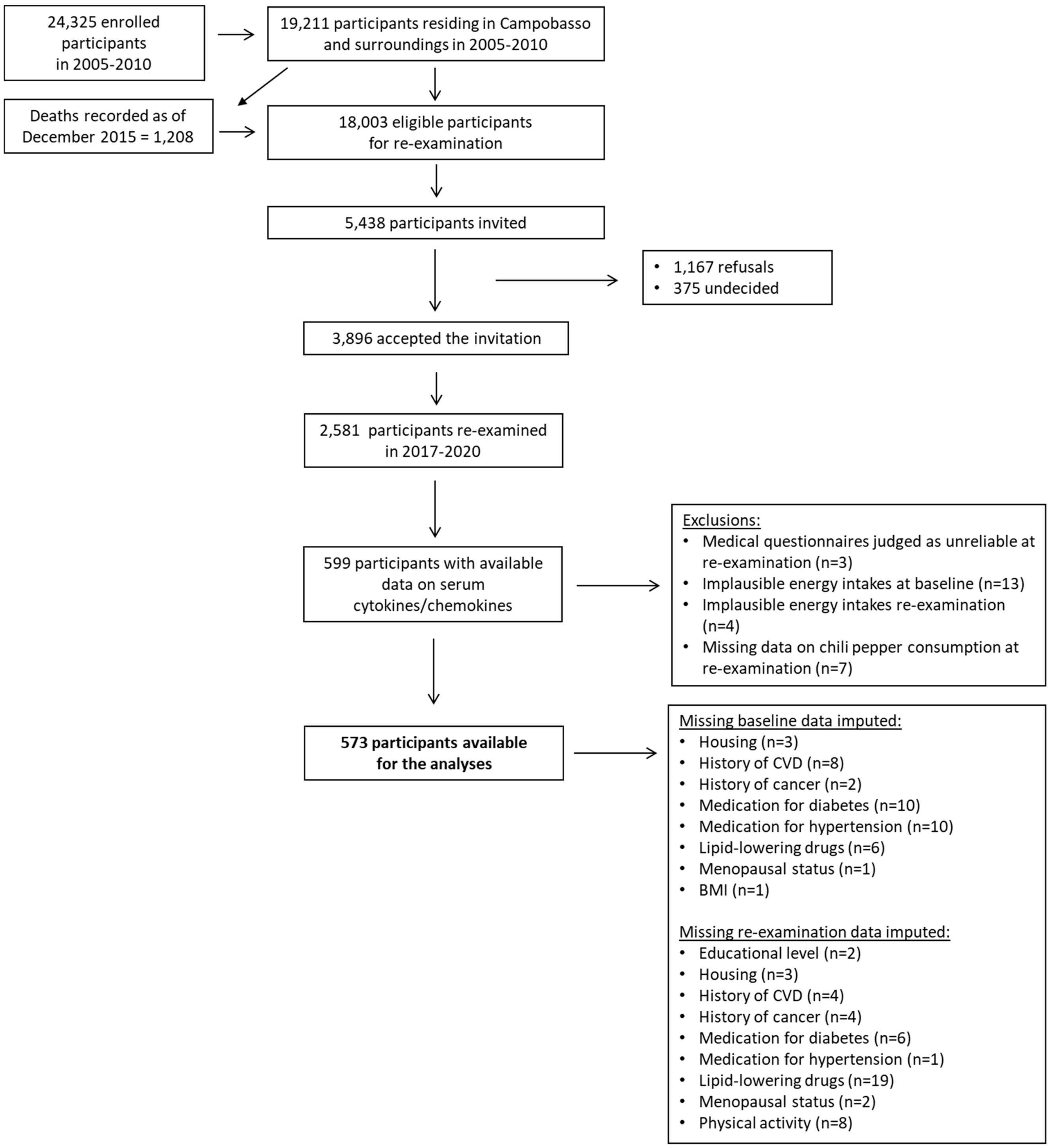

Table S1. Mean serum concentrations of analytes at baseline and re-examination

Blood markers (pg/ml)	Whole sample (n=573)		Men (n=266)		Women (n=307)	
	**Mean**	**Std Dev**	**Mean**	**Std Dev**	**Mean**	**Std Dev**
TNF-α (baseline)	6.90	8.83	7.09	3.93	6.74	11.50
TNF-α (re-examination)	8.56	9.68	8.41	5.15	8.69	12.33
Δ TNF-α	1.66	12.19	1.31	4.91	1.95	16.02
VEGF (baseline)	306.62	177.84	300.87	169.22	311.61	185.11
VEGF (re-examination)	265.28	183.24	260.27	178.17	269.63	187.70
Δ VEGF	-41.34	120.87	-40.60	134.85	-41.98	107.52
MCP-1 (baseline)	407.79	141.87	433.31	151.73	385.69	128.95
MCP-1 (re-examination)	375.95	173.64	388.42	211.41	365.14	131.92
Δ MCP-1	-31.85	149.40	-44.88	179.89	-20.55	115.84
IP-10 (baseline)	97.66	99.35	101.03	119.76	94.74	77.52
IP-10 (re-examination)	100.51	123.30	103.13	146.56	98.24	99.02
Δ IP-10	2.85	118.58	2.10	146.27	3.50	88.07
IL-6 (baseline)	5.02	12.32	5.06	7.04	4.99	15.52
IL-6 (re-examination)	6.39	14.64	5.50	4.96	7.17	19.44
Δ IL-6	1.37	17.65	0.44	7.10	2.18	23.18
IL-8 (baseline)	13.48	8.91	14.13	10.37	12.92	7.38
IL-8 (re-examination)	12.06	8.22	12.32	9.47	11.84	6.96
Δ IL-8	-1.42	10.45	-1.81	12.59	-1.08	8.15
IL-10 (baseline)	91.87	113.45	93.03	118.05	90.87	109.49
IL-10 (re-examination)	121.91	116.88	116.53	101.34	126.57	128.82
Δ IL-10	30.03	110.99	23.50	102.07	35.69	118.06
IFN-γ (baseline)	1.29	1.95	1.30	1.92	1.29	1.98
IFN-γ (re-examination)	2.31	20.16	1.31	2.19	3.17	27.46
Δ IFN-γ	1.02	19.47	0.01	2.38	1.88	26.49
CRP (baseline; mg/L)	2.15	2.76	1.94	2.75	2.34	2.76
CRP (re-examination; mg/L)	3.10	5.12	2.51	4.12	3.61	5.80
Δ CRP (mg/L)	0.95	5.31	0.57	4.55	1.27	5.88
INFLA-score (baseline)	-0.01	6.11	-0.03	6.21	0.00	6.04
INFLA-score (re-examination)	0.00	5.93	-0.01	5.70	0.01	6.14
Δ INFLA-score	0.02	5.65	0.02	5.92	0.01	5.42

TNF-α, tumor necrosis factor α; VEGF, vascular endothelial growth factor; MCP-1, monocyte chemoattractant protein-1; IP-10, interferon gamma-induced protein 10; IL-6, interleukin-6; IL-8, interleukin-8; IL-10, interleukin-10; IFN-γ, interferon-γ; CRP, C-reactive protein.

Moli-sani Study Investigators

The enrolment phase of the Moli-sani Study was conducted at the Research Laboratories of the Catholic University in Campobasso (Italy), the follow up of the Moli-sani cohort is being conducted at the Research Unit of Epidemiology and Prevention of the IRCCS Neuromed, Pozzilli, Italy.

Steering Committee: Licia Iacoviello*# (Chairperson), Giovanni de Gaetano*, Maria Benedetta Donati*.
Scientific Secretariat: Chiara Cerletti* (Coordinator), Marialaura Bonaccio*, Americo Bonanni*, Simona Costanzo*°, Amalia De Curtis*, Augusto Di Castelnuovo*, Alessandro Gialluisi*#, Francesco Gianfagna°, Mariarosaria Persichillo*†.
Safety and Ethical Committee: Jos Vermylen (Catholic University, Leuven, Belgio) (Chairperson), Renzo Pegoraro (Pontificia Accademia per la Vita, Roma, Italy), Antonio G. Spagnolo (Catholic University, Roma, Italy).
External Event Adjudicating Committee: Deodato Assanelli (Brescia, Italy), Livia Rago (Campobasso, Italy).
Baseline and Follow-up Data Management: Simona Costanzo*° (Coordinator), Teresa Panzera*.
Biobanking and Clinical Chemistry: Amalia De Curtis* (Coordinator), Sara Magnacca*.
Data Analysis: Augusto Di Castelnuovo* (Coordinator), Marialaura Bonaccio*, Simona Costanzo*°, Alessandro Gialluisi*#, Francesca Bracone*, Giuseppe Di Costanzo*, Simona Esposito*#, Martina Morelli*±, Antonietta Pepe*#, Emilia Ruggiero*#, Anwal Ghulam°, Francesco Gianfagna°, Maria Loreto Muñoz Venegas±, Sukshma Sharma±.
Recruitment Centre: Francesca Bracone* (Coordinator), Fiorella De Rita*, Giuseppe Di Costanzo*.
Communication and Press Office: Americo Bonanni*^.

External Collaboration

Genomics: Nicole Soranzo, Nicola Pirastu, Federica Santonastaso, Edoardo Giacopuzzi, Chiara Chiereghin, Human Technopole, Milan, Italy.
Epigenomics: Danilo Licastro, Simone Del Monego, Area Science Park, Trieste, Italy.

Institutions and Hospitals involved in follow-up activities: Direzione Generale per la Salute - Regione Molise; Azienda Sanitaria Regionale del Molise (ASReM, Italy); Agenzia Regionale per la Protezione Ambientale del Molise (ARPA Molise, Italy); Molise Dati Spa (Campobasso, Italy); Offices of vital statistics of the Molise Region. Presidi Ospedalieri ASReM: Ospedale A. Cardarelli -Campobasso, Ospedale F. Veneziale -Isernia, Ospedale San Timoteo -Termoli (CB), Ospedale Ss. Rosario -Venafro (IS), Ospedale Vietri -Larino (CB), Ospedale San Francesco Caracciolo -Agnone (IS); Casa di Cura Villa Maria -Campobasso; Responsible Research Hospital -Campobasso; IRCCS Neuromed -Pozzilli (IS).

Research Unit of Epidemiology and Prevention, IRCCS Neuromed, Pozzilli, Italy
#*Department of Medicine and Surgery, LUM University "Giuseppe Degennaro", Casamassima, Italy*
°*Department of Medicine and Surgery, University of Insubria, Varese, Italy*
±*Fondazione Veronesi – Piattaforma UMBERTO, Pozzilli, Italy*
^ *Multimed Comunicazioni, Pozzilli, Italy*
†*Passed away November 3rd 2025*
The list of the Moli-sani Study Past Investigators is available at https://www.moli-sani.org/?page_id=173
